# Effectiveness of diabetes and congenital adrenal hyperplasia meeting clubs

**DOI:** 10.1186/1687-9856-2013-S1-P134

**Published:** 2013-10-03

**Authors:** Quynh TV Huynh, HTD Thuy, Kate Armstrong, Maria Craig

**Affiliations:** 1 Univeristy Of Medicine And Pharmacy Ho Chi Minh City, Vietnam; 2 Director of Caring and Living as Neigbour (CLAN), Australia; 3 Children's Hospital of Westmead, Sydney, Australia

## 

Education is a vitally important component of caring for children with congenital hyperplasia and diabetes, helping families feel more confident and the children to enjoy the highest quality of life possible. At present in Vietnam, tertiary children’s hospitals are almost busy and overcrowded. Doctors have very limited time to spend with patients and their families, and outpatient visits are usually limited to about 5-10 minutes per child. To overcome these problems, Children’s Hospital 2 (CH2 - in Ho Chi Minh City) in collaboration with CLAN (Caring and Living As Neighbour Organization) and Children’s Hospital Westmead (CHW) has been holding annual Club meetings for all diabetes and congenital adrenal hyperplasia (CAH) patients and families since 2009. These Clubs are an opportunity for health professionals to educate families and for families to meet and interact.

The study aimed to answer 3 questions:

1) Perceived benefits of the Clubs

2) Changes to management as a result of attending the Clubs

3) Recommendations on ways to improve the Clubs

A survey of families (written format with closed and open questions) attending the 2011 CAH and Diabetes Club meetings was undertaken at CH2 Outpatient Clinic visits 6 months after the Club meeting.

29/31 diabetes families (93,5%) and 18/19 CAH families (94%) were surveyed. 100% of parents indicated they felt their overall knowledge had improved. Amongst the diabetes families: 50% felt more confident with sick day management; 48% reported changes in diets and improved blood glucose control (48%); 20% visited the hospital more frequently for complication screening; and 10% reported prompting doctors to screen for complication when they felt this had been forgotten. Of the CAH’s families: 50% felt more confident with sick day management and increasing dose of hydrocortisone independently; 36% let children go to the kindergarten; 29% began letting children play sports; 29% of mothers had attended genetic consultants for the next pregnancy; and 7% of children had benefited from surgery that the families had previously been too afraid to undertake.The majority of families suggested the club meetings should be longer and focus more on specific areas of interest: nutrition, exercise and physiology in diabetes and physiology, genetic consulting in CAH.

Families reported significant benefits from attending CAH and Diabetes Clubs, with some substantial improvements in quality of life for the children.

